# Clinical spectrum of *Transthyretin* amyloidogenic mutations among diverse population origins

**DOI:** 10.1186/s40246-024-00596-7

**Published:** 2024-03-25

**Authors:** Antonella De Lillo, Gita A. Pathak, Aislinn Low, Flavio De Angelis, Sarah Abou Alaiwi, Edward J. Miller, Maria Fuciarelli, Renato Polimanti

**Affiliations:** 1 Department of Psychiatry, Yale University School of Medicine, 60 Temple, Suite 7A, New Haven, CT 06510 USA; 2 Department of Biology, University of Rome “Tor Vergata”, Rome, Italy; 3 VA CT Healthcare Center, West Haven, CT USA; 4 Department of Physical and Mental Health, and Preventive Medicine, University of Campania “Luigi Vanvitelli”, Naples, Italy; 5 Section of Cardiovascular Medicine, Department of Internal Medicine, Yale University School of Medicine, New Haven, CT USA; 6 Wu Tsai Institute, Yale University, New Haven, CT USA

**Keywords:** TTR amyloidosis, Electronic health records, Diversity, Disease associations

## Abstract

**Purpose:**

Coding mutations in the *Transthyretin* (*TTR*) gene cause a hereditary form of amyloidosis characterized by a complex genotype-phenotype correlation with limited information regarding differences among worldwide populations.

**Methods:**

We compared 676 diverse individuals carrying *TTR* amyloidogenic mutations (rs138065384, Phe44Leu; rs730881165, Ala81Thr; rs121918074, His90Asn; rs76992529, Val122Ile) to 12,430 non-carriers matched by age, sex, and genetically-inferred ancestry to assess their clinical presentations across 1,693 outcomes derived from electronic health records in UK biobank.

**Results:**

In individuals of African descent (AFR), Val122Ile mutation was linked to multiple outcomes related to the circulatory system (fold-enrichment = 2.96, *p* = 0.002) with the strongest associations being cardiac congenital anomalies (phecode 747.1, *p* = 0.003), endocarditis (phecode 420.3, *p* = 0.006), and cardiomyopathy (phecode 425, *p* = 0.007). In individuals of Central-South Asian descent (CSA), His90Asn mutation was associated with dermatologic outcomes (fold-enrichment = 28, *p* = 0.001). The same *TTR* mutation was linked to neoplasms in European-descent individuals (EUR, fold-enrichment = 3.09, *p* = 0.003). In EUR, Ala81Thr showed multiple associations with respiratory outcomes related (fold-enrichment = 3.61, *p* = 0.002), but the strongest association was with atrioventricular block (phecode 426.2, *p* = 2.81 × 10^− 4^). Additionally, the same mutation in East Asians (EAS) showed associations with endocrine-metabolic traits (fold-enrichment = 4.47, *p* = 0.003). In the cross-ancestry meta-analysis, Val122Ile mutation was associated with peripheral nerve disorders (phecode 351, *p* = 0.004) in addition to cardiac congenital anomalies (fold-enrichment = 6.94, *p* = 0.003).

**Conclusions:**

Overall, these findings highlight that *TTR* amyloidogenic mutations present ancestry-specific and ancestry-convergent associations related to a range of health domains. This supports the need to increase awareness regarding the range of outcomes associated with *TTR* mutations across worldwide populations to reduce misdiagnosis and delayed diagnosis of TTR-related amyloidosis.

**Supplementary Information:**

The online version contains supplementary material available at 10.1186/s40246-024-00596-7.

## Introduction

Transthyretin (TTR) is a homo-tetrameric protein involved in a wide range of physiological processes [1, 2]. The dissociation of the TTR tetramer initiates an amyloidogenic process, where the misfolded monomers lead to non-native oligomers and finally amyloid fibrils [3]. Coding mutations in the *TTR* gene cause TTR misfolding and are responsible for a hereditary form of amyloidosis, TTR-related amyloidosis (hATTR) [4]. TTR amyloid fibrils can affect a range of tissues and organs, resulting in a high clinical variability among individuals carrying *TTR* amyloidogenic mutations [5, 6]. While hATTR is an autosomal dominant disease, penetrance is variable, and patients can present with distinct phenotypes and differential organ involvement including cardiomyopathy, polyneuropathy, vasculopathy, gastrointestinal impairment, nephropathy, ocular deposition, and genitourinary involvement [7–11]. hATTR is often misdiagnosed or diagnosed several years after the onset of the initial symptoms due to the heterogeneous clinical presentation of *TTR* amyloidogenic mutations^12,13^. A consistent variation in hATTR clinical presentation has been also reported among different population origins [5, 6, 8]. However, population comparisons have mostly been limited to cohorts of European and East Asian descent for most *TTR* amyloidogenic mutations (e.g., *TTR* Val30Met, rs28933979) and to individuals of African descent for *TTR* Val122Ile mutation (rs76992529) [8, 10, 12]. Recently, molecular and computational studies demonstrated that non-coding mechanisms regulating transcriptomic and epigenetic variation of the *TTR* gene partially explain hATTR clinical heterogeneity [13–16], including the differences among diverse populations [17–19]. However, the knowledge of the clinical presentation associated with *TTR* amyloidogenic mutations is still too limited for several human populations. This can also lead to a lack of awareness among clinicians in areas that do not have a previous history of hATTR cases.

To expand the understanding of the clinical spectrum of *TTR* mutations among underrepresented, diverse ancestral groups, we performed a phenome-wide analysis comparing *TTR* mutations in individuals of African (AFR), Admixed-American (AMR), Central/South Asian (CSA), East Asian (EAS), and Middle Eastern (MID) descents to non-carriers matched for age, sex, and genetically-inferred ancestry. Specifically, we systematically tested associations across 1,693 outcomes derived from electronic health records available from the UK Biobank (UKB) [20] and investigated ancestry-specific and ancestry-convergent enrichment for health domains and comorbidity networks linking TTR-associated outcomes.

## Materials and methods

### Study population

The UKB cohort is a controlled access genomics resource containing information on a wide range of illnesses as well as normal-range traits [20]. This project has recruited more than 500,000 people. Phenotypic information was collected for each participant using multiple data sources. In the present study, we derived clinical information regarding a range of health outcomes from UKB electronic health records. Specifically, we used previously defined phecode categorization [21] to translate the International Classification of Diseases 9th and 10th (ICD9/ICD10) codes into clinically meaningful phecode outcomes. A total of 1,693 phecodes were available from UK Biobank (Supplemental Table 1). UKB phecodes are grouped into 17 categories: circulatory system (*N* = 155), congenital anomalies (*N* = 55), dermatologic (*N* = 95), digestive (*N* = 155), endocrine/metabolic (*N* = 151), genitourinary (*N* = 156), hematopoietic (*N* = 54), infectious diseases (*N* = 58), injuries & poisonings (*N* = 133), mental disorders (*N* = 71), musculoskeletal (*N* = 122), neoplasms (*N* = 135), neurological (*N* = 78), pregnancy complications (*N* = 49), respiratory (*N* = 75), sense organs (*N* = 113), and symptoms (*N* = 38).

Genome-wide genotyping was performed on all UKB participants using the UKB Axiom Array, with approximately 850,000 variants directly measured and > 90 million variants imputed using the Haplotype Reference Consortium and UK10K + 1000 Genomes Project reference panels [20]. Pan-UKB investigators used genome-wide data to infer the ancestral backgrounds of UKB participants. Details are available at https://pan.ukbb.broadinstitute.org/docs/qc. Briefly, ancestries (i.e., AFR, AMR, CSA, EAS, EUR, and MID) were assigned using a random forest classifier based on features predictive of similarities with reference populations derived from a combined 1,000 Genomes Project [22] and Human Genome Diversity Panel [23]. Considering Pan-UKB ancestry assignments and genetically unrelated individuals (kinship coefficients < 0.125), we identified 676 individuals carrying four different *TTR* mutations (rs138065384, Phe44Leu, p.Phe64Leu; rs730881165, Ala81Thr, p.Ala101Thr; rs121918074, His90Asn, p.His110Asn.; rs76992529, Val122Ile, p.Val142Ile; Table 1). No other potentially pathogenic *TTR* mutation was identified in UKB cohort. Phe44Leu, Ala81Thr, and Val122Ile were classified as amyloidogenic (Supplemental Table 2) according to a curated database [24] available at http://www.amyloidosismutations.com/. This online registry aims to collect information on mutations associated with hereditary amyloidosis, providing details regarding their pathogenicity based on (i) sufficient clinical information, (ii) histological evidence of amyloid deposition, and (iii) appropriate amyloid fibril type [24]. In addition to the three mutations, we also considered His90Asn, because of the recent evidence supporting its pathogenicity [25]. Considering ClinVar germline classification [26] performed according to the criteria recommended by the American College of Medical Genetics and Genomics and the Association for Molecular Pathology (ACMG/AMP) [27], Val122Ile is classified as pathogenic, while Phe44Leu, Ala81Thr, His90Asn are reported as “Conflicting classifications of pathogenicity” (Supplemental Table 2). High-quality genotype data were available for Phe44Leu, Ala81Thr, His90Asn, and Val122Ile in UKB (INFO > 0.6; Supplemental Table 2). The minor allele frequency of these variants was in line with what reported in the reference populations available from the 1,000 Genomes Project [22] and the Genome Aggregation Database [28] (Supplemental Table 3). To define genetically comparable non-carrier group, we used MatchIT R package [29], considering age, sex, and the first ten within-ancestry principal components as matching criteria. To achieve the best balance in our dataset, we used the “nearest” method that performs greedy nearest neighbor matching by computing a distance between carrier and non-carrier samples. The optimal number of matched non-carriers to each carrier was defined using a k:1 matching. Chi-square and t-test analyses were performed to compare carriers and non-carriers with respect to binary and quantitative matching criteria, respectively. No statistical difference (*p* > 0.1) was observed for age, sex, and within-ancestry principal components between carriers and matched non-carriers in each of the ancestry-mutation groups investigated (Supplemental Table 4).

**Table 1:** TTR amyloidogenic mutations and matched controls from UKB. Information about ancestral background, age, sex, number of individuals carrying TTR amyloidogenic mutations (N_carriers_) and non-carriers (N_non−carriers_) for each population, and the total number of individuals are reported. AFR: African; AMR: admixed Americans; CSA: Central-South Asian; EAS: East Asian; EUR: European; MID: Middle Eastern

Mutation (rsid)	Ancestry	Age, mean (SE)	Female, %	N_carriers_	N_non−carriers_
Phe44Leu (rs138065384)	AFR	58.8 (0.43)	100	2	200
Ala81Thr (rs730881165)	EAS	49 (0.32)	28	4	400
	EUR	56.8 (0.12)	55	347	3,470
His90Asn (rs121918074)	CSA	53 (0.86)	35	1	100
	EUR	57 (0.14)	53	33	3,300
	MID	54.3 (0.79)	100	1	100
Val122Ile (rs76992529)	AFR	52.8(0.15)	54	266	2,660
	AMR	54.5 (0.88)	100	1	100
	CSA	59.7(0.50)	0	2	200
	EUR	52.3 (0.21)	46	15	1,500
	MID	45.9 (0.24)	55	4	400

### Statistical analyses

To investigate the association of *TTR* amyloidogenic mutations (Phe44Leu, Ala81Thr, His90Asn, and Val122Ile; Supplemental Table 2) with clinically meaningful phenotypes (Supplemental Table 1), we compared carrier group and matched non-carriers using Fisher’s exact test. This approach was chosen because it is robust to investigate genetic associations when there is an extreme case-control imbalance due to low minor allele frequency and/or to the rare conditions investigated [30]. In the present study, we used *fisher.test* R function, analyzing ancestry-mutation groups separately (Table 1). A cross-ancestry meta-analysis was performed to investigate ancestry-convergent associations of *TTR* amyloidogenic mutations. This analysis was conducted using the sample-size weighted approach available in METAL [31]. Specifically, we converted Fisher’s exact test ancestry-specific *p* values into signed z scores where positive values correspond to positive associations between *TTR* mutations and the phecodes and negative values correspond to negative associations. With the METAL sample-size weighted approach [31], the ancestry-specific z scores were combined across samples with weights proportional to the square-root of the effective sample size. The effective sample size was calculated with the following formula: $4/(1/{N}_{cases}+1/{N}_{controls})$. Heterogeneity statistics (I^2^, chi-square, and *p* values) were calculated to estimate the variation among ancestry-specific effects. While all ancestry-specific effects were entered in the cross-ancestry meta-analysis, this was limited to phecodes with at least two ancestry-specific z scores≠0.

To test the over-representation of categories among the associations between *TTR* mutations and the phecodes investigated, we calculated the statistical significance of the phenotypic enrichment. Considering the hypergeometric cumulative distribution function, we compared the proportions of the phenotypic classes associated with the variants investigated with the proportion of phenotypic classes across the overall phenotypic spectrum investigated for each ancestry-mutation group. Over-representation analysis was also conducted with respect to cross-ancestry associations observed for each mutation.

Because the limited sample size available did not provide adequate statistical power to test individual phecode associations applying a stringent multiple testing correction, the primary outcomes of the study were the category enrichments surviving false discovery rate (FDR q < 0.05) accounting for the number of category enrichments tested. Within each FDR-significant category, we prioritized the top phecode associations.

### Comorbidity analysis

To investigate comorbidities among clinical outcomes associated with *TTR* mutations, we used previously calculated UK Biobank data [32]. Specifically, we considered information regarding relative risk (RR) to measure the tendency of co-occurrence among disease pairs. The RR for two diseases was measured as the ratio of risk between the diseases based on the disease prevalence. RR > 1 indicates a co-occurrence among the disease pair tested, while RR < 1 corresponds to a less probable co-occurrence. Bonferroni correction was applied to account for the number of disease pairs tested.

## Results

In UKB cohorts, we identified four *TTR* amyloidogenic mutations: Phe44Leu, Ala81Thr, His90Asn, and Val122Ile (Table 1). While Phe44Leu was observed only in AFR individuals, the other mutations were observed in multiple ancestry groups. Specifically, Val122Ile was identified in AFR, AMR, CSA, EUR, and MID; His90Asn in CSA, EUR, and MID; Ala81Thr in EAS and EUR. Ancestry-specific allele frequency of these mutations was in line with that observed in the reference populations available from the 1,000 Genomes Project [22] and the Genome Aggregation Database [28] (Supplemental Table 3).

**Fig. 1:**
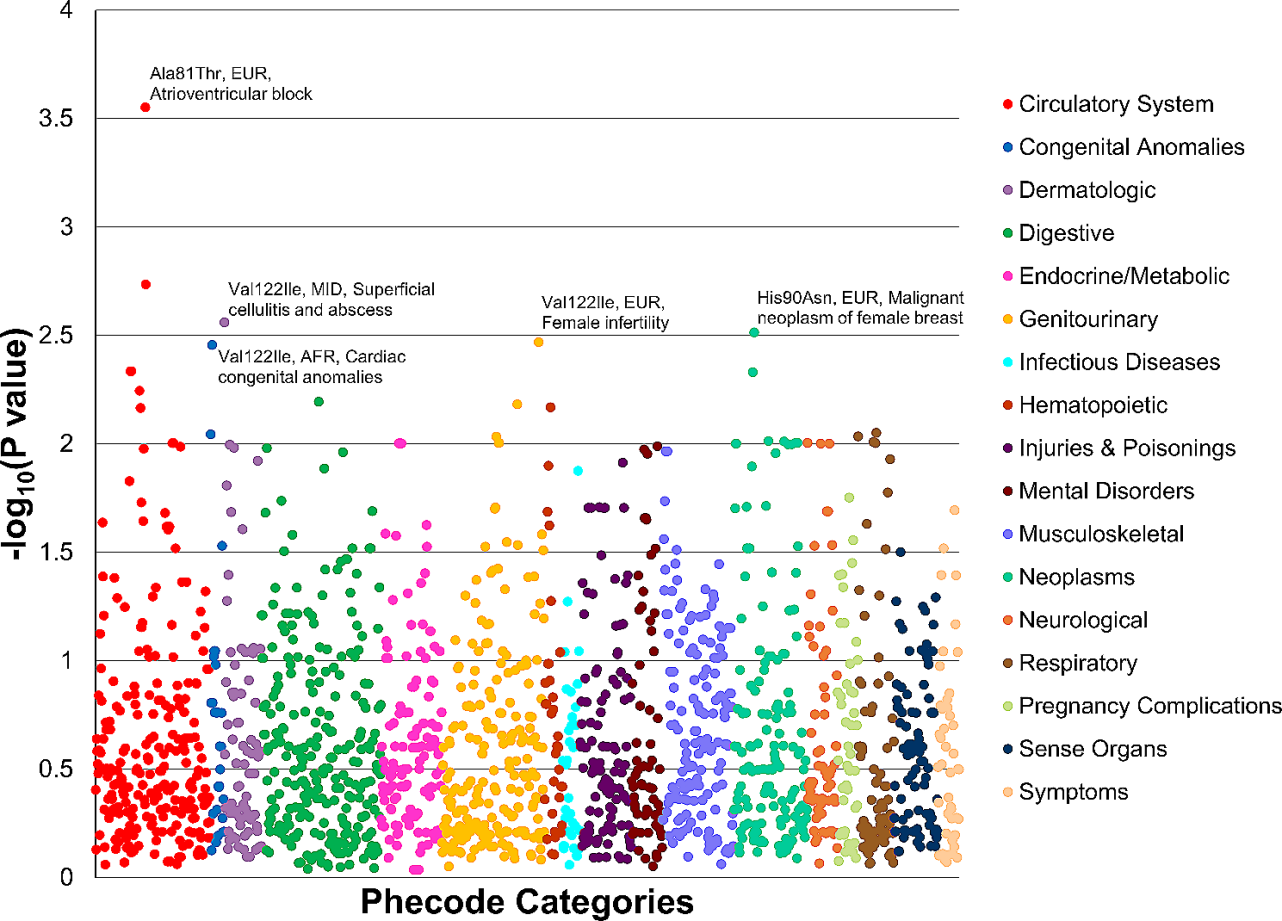
Phenome-wide associations of *TTR* amyloidogenic mutations across ancestry groups. Plotted results include those with *p* < 1. Full results are available in Supplemental Table 5. AFR: African; EUR: European; MID: Middle Eastern

In our phenome-wide analysis, we identified a total of 179 nominally significant associations (*p* < 0.05; Fig. 1, Supplemental Table 5) with respect to *TTR* Ala81Thr, His90Asn, and Val122Ile mutations (60, 54, and 65 associations, respectively). No associations were observed with respect to *TTR* Phe44Leu variant (*p* > 0.05; Supplemental Table 5). Considering phecode categories, we observed statistically significant overrepresentation of associations across the mutation-by-ancestry analyses (Table 2; Supplemental Table 6). Five of them survived FDR multiple testing correction (FDR q < 0.05). Because no phecode reached a nominally significant association with respect to Phe44Leu, we did not perform an overrepresentation test for this mutation. In AFR, Val122Ile associations related to circulatory system were significantly enriched (fold-enrichment = 2.96, *p* = 0.002). In this category, the strongest AFR-Val122Ile associations were endocarditis (phecode 420.3, *p* = 0.006) and cardiomyopathy (phecode 425, *p* = 0.007). Although they were included among congenital anomalies, Val122Ile in AFR was also associated with cardiac congenital anomalies (phecode 747.1, *p* = 0.003). No category enrichment survived multiple testing correction for Val122Ile associations in the other ancestry groups (FDR q > 0.05). Conversely, multiple ancestry-specific enrichments for Ala81Thr and His90Asn associations survived FDR multiple testing correction (Table 2). With respect to Ala81Thr mutations, EAS showed an overrepresentation of endocrine/metabolic associations (fold-enrichment = 4.47, *p* = 0.003) such as pituitary hyperfunction (phecode 253.1, *p* = 0.01) and hypoosmolality/hyponatremia (phecode 276.12, *p* = 0.03). In EUR, Ala81Thr mutation was enriched for respiratory associations (fold-enrichment = 3.61, *p* = 0.002) such as obstructive chronic bronchitis (phecode 496.21, *p* = 0.009). However, the strongest EUR-Ala81Thr association was with atrioventricular block (phecode 426.2, *p* = 2.81 × 10^− 4^) although the overrepresentation for circulatory associations was only nominally significant (fold-enrichment = 2.47, *p* = 0.005, FDR q = 0.056). With respect to His90Asn mutation, CSA was associated with dermatologic outcomes (fold-enrichment = 28, *p* = 0.001), such as changes in skin texture (phecode 687.3, *p* = 0.01). In the EUR-His90Asn analysis, we observed an overrepresentation of associations related to neoplasms category (fold-enrichment = 3.09, *p* = 0.003). These included neoplasms located in different body sites such as malignant neoplasm of female breast (phecode 174.11, *p* = 0.003), malignant neoplasm of other urinary organs (phecode 189.4, *p* = 0.01), and neurofibromatosis (phecode 199.4, *p* = 0.01).

**Table 2:** Enrichment analysis of health categories with respect to phecodes investigated across TTR mutations and ancestry groups. Expected phecodes are those that would have been associated in case of no enrichment. Overrepresentation tests were conducted with respect to ancestry-specific associations reaching nominal significance. Nominally significant results (*p* < 0.05) are reported. Findings surviving false discovery rate multiple testing correction (FDR q < 0.05) are indicated in bold. Details of all categories tested are available in Supplemental Table 6. AFR: African; AMR: Admixed-American; Central-South Asian; EAS: East Asian; EUR: European; MID: Middle Eastern

Mutation (rsID)	Ancestry	Category	Phecodes associated in the category, N	Expected phecodes associated in the category, N	Fold-Enrichment	*P*	FDR q
Ala81Thr (rs730881165)	EAS	**Endocrine/Metabolic**	**5**	**1**	**4.47**	**0.003**	**0.040**
		Neurological	3	1	3.80	0.040	0.168
		Pregnancy complications	3	1	5.07	0.018	0.124
	EUR	Circulatory system	10	4	2.47	0.005	0.056
		Mental disorders	5	2	2.89	0.026	0.124
		**Respiratory**	**7**	**2**	**3.61**	**0.002**	**0.040**
His90Asn (rs121918074)	CSA	**Dermatologic**	**2**	**0**	**28.00**	**0.001**	**0.040**
	EUR	**Neoplasms**	**8**	**3**	**3.09**	**0.003**	**0.040**
	MID	Digestive	8	4	2.15	0.021	0.124
		Mental disorders	3	0	6.68	0.008	0.077
		Symptoms	3	1	4.45	0.025	0.124
Val122Ile (rs76992529)	AFR	**Circulatory system**	**9**	**3**	**2.96**	**0.002**	**0.040**
		Hematopoietic	4	1	4.44	0.011	0.092
	AMR	Neplasms	2	0	7.78	0.020	0.124
	CSA	Genitourinary	3	1	4.07	0.031	0.138
	MID	Genitourinary	4	1	3.28	0.024	0.124

In the cross-ancestry meta-analysis (Supplemental Table 7), the strongest association was between Val122Ile and other peripheral nerve disorders (phecode 351, *p* = 0.004). No cross-ancestry heterogeneity (heterogeneity *p* = 0.693) was observed in the ancestry-specific effects in AFR and EUR (AFR *p* = 0.029; EUR *p* = 0.059). With respect to Ala81Thr mutation, the strongest cross-ancestry association was observed with respect to dysphagia (phecode 532, *p* = 0.007; cross-ancestry heterogeneity *p* = 0.362) where consistent effects were observed in EUR and EAS (EUR *p* = 0.026; EAS *p* = 0.08). Considering category overrepresentation (Supplemental Table 8), Val122Ile mutation was enriched for congenital anomalies (fold-enrichment = 6.94, *p* = 0.003) with the strongest evidence observed with respect to cardiac congenital anomalies (phecode 747.1, cross-ancestry *p* = 0.005). Although there was no statistically significant heterogeneity (heterogeneity *p* = 0.110), the cross-ancestry association was primarily driven by the effect observed in AFR (AFR *p* = 0.003), which was much stronger than the one in EUR (EUR *p* = 0.156). Considering cross-ancestry heterogeneity statistics, we observed an overrepresentation of nominally significant heterogeneous effects of Val122Ile among neoplasm-related outcomes (fold-enrichment = 4.53, *p* = 0.005). The strongest evidence was related to lipoma (phecode 214, cross-ancestry heterogeneity *p* = 0.006) where AFR and AMR Val122Ile associations showed opposite effect directions (AFR *p* = 0.077, direction +; AMR *p* = 0.010, direction -).

**Fig. 2:**
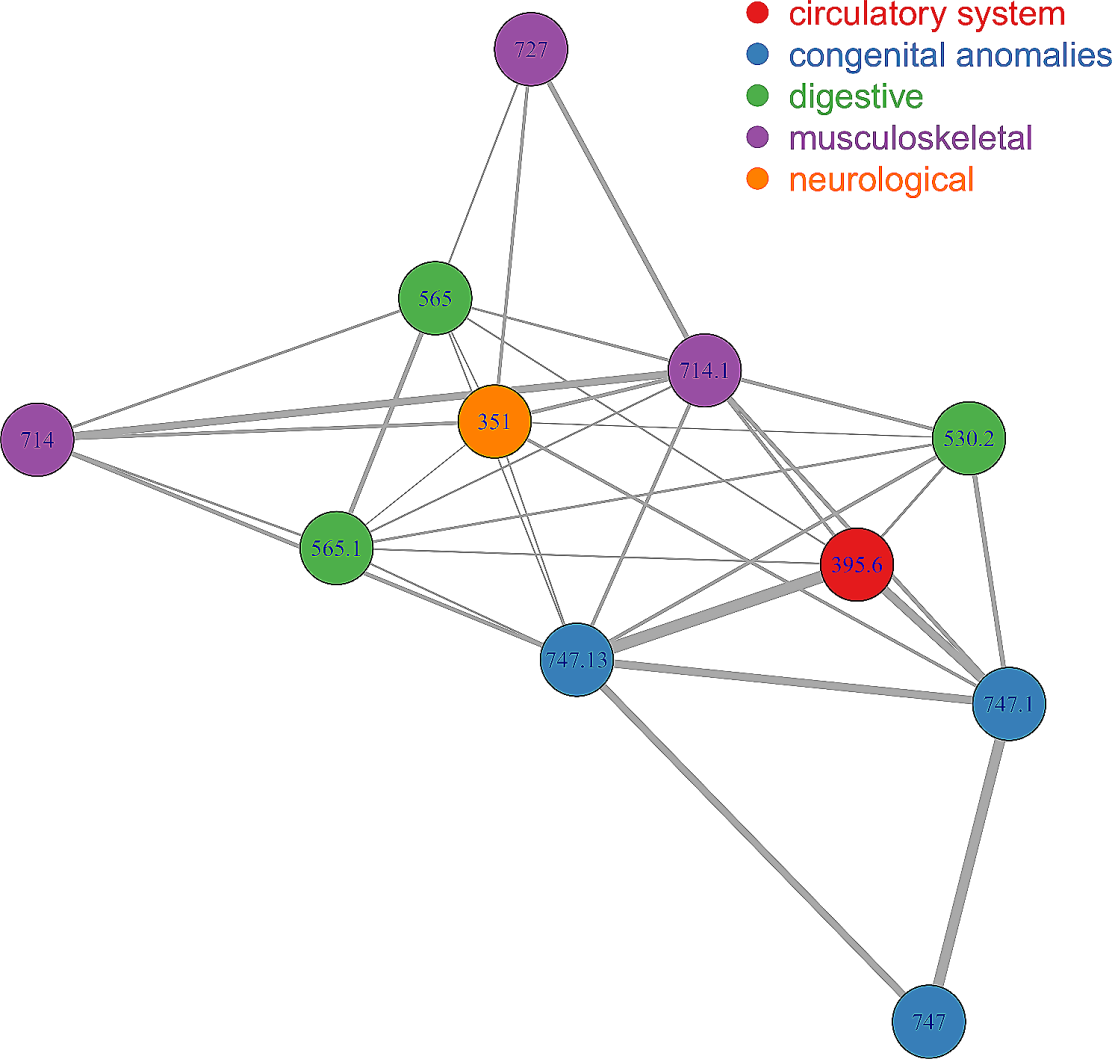
Comorbidity network of phecodes associated with *TTR* Val122Ile mutation in the cross-ancestry meta-analysis. Edges are proportional to the relative risk of pair-wise comorbid phecodes surviving Bonferroni multiple testing correction (*p* < 8.62×10^− 4^). Full results are available in Supplemental Table 9. 351: Other peripheral nerve disorders; 395.6: Heart valve replaced; 530.2: Esophageal bleeding (varices_hemorrhage); 565: Anal and rectal conditions; 565.1: Anal and rectal polyp; 714: Rheumatoid arthritis and other inflammatory polyarthropathies; 714.1: Rheumatoid arthritis; 727: Other disorders of synovium, tendon, and bursa; 747: Cardiac and circulatory congenital anomalies; 747.1: Cardiac congenital anomalies: 747.13: Congenital anomalies of great vessels

Because multiple phecodes were associated with Val122Ile mutation in the cross-ancestry meta-analysis (Supplemental Table 7), we investigated their comorbidity network. This analysis was limited to 11 phecodes with comorbidity data available [32]. Utilizing a Bonferroni correction to account for the number of pair-wise comparisons tested (*p* < 8.62×10^− 4^), we identified 35 statistically significant comorbid pairs (Fig. 2; Supplemental Table 9). Twenty-seven of them were pairs of phecodes related to different categories. Among these cross-category pairs, the highest RR estimates were observed for the comorbidity of cardiac congenital anomalies (phecode 747.1) with heart valve replaced (phecode 395.6, circulatory category; RR = 20.29, *p* = 2.34×10^− 114^), esophageal bleeding (phecode 530.2, digestive category; RR = 5.14, *p* = 2.20×10^− 7^), and rheumatoid arthritis (phecode 714.1, musculoskeletal category; RR = 4.18, *p* = 7.89×10^− 7^).

## Discussion

The present study leveraged genetic and clinical data from UKB cohort to understand the spectrum of health outcomes associated with *TTR* amyloidogenic mutations among diverse ancestral backgrounds. Several studies highlighted differences in the genotype-phenotype correlation of *TTR* mutations among human populations [8, 10, 12]. However, there are still several ancestry groups where no information is available regarding *TTR* mutations. In the present study, we broaden the knowledge regarding the distribution and the clinical spectrum of *TTR* amyloidogenic mutations in six ancestry groups. For example, *TTR* Val122Ile mutation is well-known to affect primarily AFR individuals, but it can be present also in EUR populations due to an independent mutation event rather than genetic admixture [33, 34]. In UKB, Val122Ile was identified in unrelated participants of AFR, AMR, CSA, EUR, and MID descent. While Val122Ile mutation has been previously reported in Hispanic and Latino America populations [35], very limited information is available regarding hATTR in CSA and MID individuals [36, 37]. In line with the known causal role of Val122Ile in cardiac amyloidosis [38], we observed an overrepresentation of Val122Ile associations with circulatory phecodes in AFR. One of the strongest findings was related to cardiomyopathy, which is a primary Val122Ile outcome due to the accumulation of TTR fibrils in the myocardium [39]. We also observed that Val122Ile mutation in AFR could be linked to endocarditis (phecode 420.3). While there is no previous information regarding endocarditis and Val122Ile, this association may be related to endocarditis risk observed after pacemaker implantation [40], which is recommended for patients with cardiac amyloidosis to prevent bradycardia and syncope [41]. In the cross-ancestry meta-analysis, Val122Ile was enriched for associations with cardiac congenital anomalies with consistent effects in both AFR and EUR. Similarly, we also observed an AFR-EUR cross-ancestry association between Val122Ile mutation and peripheral nerve disorders (phecode 351). While cardiac amyloidosis is the primary Val122Ile outcome [39], our finding is consistent with previous reports supporting that Val122Ile may be related to peripheral neuropathy symptoms that are more often reported with respect to other *TTR* amyloidogenic mutations [42]. This is in line with the results of a previous phenome-wide analysis in UKB-AFR participants, reporting an association of Val122Ile with polyneuropathy [43]. The comorbidity analysis highlighted high RR among Val122Ile-associated phecodes, also when considering the whole UKB cohort. Indeed, while congenital anomalies (cardiac and of great vessels) and heart valve replacement showed a RR about four times larger than the other cross-category pairs, 16 of the 26 cross-category pairs had a RR > 2. This suggests that genetic testing remain a primary tool to distinguish comorbidity patterns due to Val122Ile.

Ala81Thr was identified in EAS and EUR participants enrolled in UKB cohort. Very limited information is available regarding this *TTR* amyloidogenic mutation, which has been mostly reported in EUR populations [44–46]. In EAS, Ala81Thr showed multiple associations with endocrine/metabolic outcomes. In this category, the most significant evidence was related to phecodes linked to neurohypophysis disorders and hypoosmolality/hyponatremia. Hyponatremia due to altered neurohypophysis regulation of vasopressin is a common electrolyte imbalance in neurologic patients [47]. In the context of hATTR, hyponatremia has been observed in cases of cardiac amyloidosis [48, 49]. Further studies will be needed to confirm and understand Ala81Thr link with neurohypophysis disorders and hypoosmolality/hyponatremia in EAS carriers. In EUR, Ala81Thr presented associations with respiratory outcomes such as obstructive chronic bronchitis. This is in line with cases of pulmonary amyloidosis reported among hATTR patients [50, 51]. Additionally, the strongest Ala81Thr association was with atrioventricular block, which is observed in 40% of patients with hATTR-cardiac amyloidosis [52]. These findings contribute to our understanding of the spectrum of Ala81Thr mutation, which is currently unclear in the current literature.

We identified *TTR* His90Asn mutation in CSA, EUR, and MID. Although this variant was observed in EUR patients with cardiac amyloidosis and amyloidotic polyneuropathy [25, 53–56], it has been previously classified as a variant of uncertain significance according to the criteria of the Dilated Cardiomyopathy Precision Medicine Study [57]. In CSA, His90Asn was enriched for associations with dermatological outcomes. These were related to changes in skin texture. Cutaneous manifestations of hATTR have been previously reported [58] and skin biopsies have been previously proposed as a potential biomarker of disease severity in affected patients [59]. As mentioned above, very limited information is available regarding the presentation of *TTR* mutations in CSA populations [37]. Our findings suggest that His90Asn is associated with dermatologic presentations in CSA. In EUR, His90Asn was associated with neoplasms at different body sites (e.g., breast, urinary organs, and neurofibromatosis). There is no literature regarding the relationship between hATTR and cancer risk. Because TTR protein has been linked to tumor growth and severity [60–63], His90Asn associations observed in our study may be related to the effect of this mutation on TTR function rather than on the amyloidogenic cascade. This is in line with the associations observed for *TTR* non-coding variants in a previous phenome-wide analysis [15]. The lack of His90Asn enrichment for well-known hATTR-related outcomes may support the uncertain amyloidogenicity of His90Asn mutation.

Although our study provided novel information regarding the clinical spectrum of *TTR* mutations across multiple ancestry groups, our analyses were based on UK Biobank, which is a cohort that largely overrepresents EUR participants. Because this is a general-population cohort from UKB, we identified only a limited number of *TTR* mutations. To account for the high case-control imbalance, we applied Fisher’s exact test which is a highly conservative approach to investigate genetic associations [30]. This may have limited the power of our association analysis. While FDR multiple testing correction was applied to our overrepresentation analyses of health categories, we discussed nominally significant phecode associations within the FDR-significant categories. Further studies will be needed to confirm the associations identified in our study. Finally, we observed only a trend-association of Val122Ile with amyloidosis phecode (270.33) in AFR (*p* = 0.068). This may be due to amyloidosis referring to a heterogeneous group of disorders [64] or to TTR amyloidosis being underdiagnosed [65].

In conclusion, the present study provided novel insights regarding the possible associations of *TTR* amyloidogenic mutations across human populations. In particular, we identified *TTR* mutations in ancestries that are underrepresented in research and literature. Our findings also highlighted differences in hATTR-related health outcomes observed among diverse ancestral backgrounds. This contributes to expand our understanding of the spectrum of clinical outcomes potentially associated with *TTR* mutations and confirms the need to increase awareness regarding hATTR to reduce misdiagnosis and delayed diagnosis due to the complex genotype-phenotype correlation of the disease.

### Electronic supplementary material

Below is the link to the electronic supplementary material.

Supplementary Material 1

Supplementary Material 2

Supplementary Material 3

Supplementary Material 4

Supplementary Material 5

Supplementary Material 6

Supplementary Material 7

Supplementary Material 8

Supplementary Material 9

## Data Availability

No datasets were generated or analysed during the current study.

## Abbreviations

AFR: African
AMR: Admixed American
CSA: Central South Asian
EAS: East Asian
EUR: European
hATTR: Hereditary transthyretin amyloidosis
MID: Middle Eastern
RR: Relative Risk
TTR: Transthyretin
UKB: UK Biobank
